# Multicolor Emitting N-Doped Carbon Dots Derived from Ascorbic Acid and Phenylenediamine Precursors

**DOI:** 10.1186/s11671-020-03453-3

**Published:** 2020-12-03

**Authors:** Linlin Wang, Won Mook Choi, Jin Suk Chung, Seung Hyun Hur

**Affiliations:** School of Chemical Engineering, University of Ulsan, Daehak-ro 93, Nam-gu, Ulsan, 44610 Republic of Korea

**Keywords:** Multicolor emission, N-doped carbon dots, Ascorbic acid, Phenylenediamine, Hydrothermal

## Abstract

In this research, we report the green, blue, and orange color emitting N-doped carbon dots (CDs), which are being synthesized from ascorbic acid and *o*-/*m*-/*p*-phenylenediamine (*o*-PDA, *m*-PDA, and *p*-PDA, respectively). The effects of the solvent polarity and solution pH on the PL emission properties of the as-synthesized CDs have been systematically investigated. It has been observed that the PL emission of the as-synthesized CDs decreases with the increase in solvent polarity due to the greater agglomeration. The surface charge of CDs also shows prominent effects on the pH-dependent PL emission properties.

## Introduction

Recently, fluorescent carbon dots (CDs) have drawn considerable attention owing to their high quantum yields, low toxicity, excellent biocompatibility, and facile preparation procedures [1–4]. CDs can be widely used in sensing, display, and bioimaging applications. Most of CDs emit in blue or green region that limits their application in living tissue imaging since this process needs deep penetration of light and removal of autofluorescence as well as background light scattering related limitations. Henceforth, synthesis of CDs that emit at larger wavelength has become important. In this regard, green chemical synthesis of multi-color emissive CDs is important that would exclude the associated synthetic hazards and critical separation steps [5].

Adjusting the surface of CDs by doping of hetero atoms, such as nitrogen (N), boron (B), and sulfur (S) atoms, can be used to modify the fluorescence properties of CDs. For this purpose, organic/inorganic molecules with hetero-atom functionalities might be used as co-precursor along with the carbon source or as precursor [6–8]. Phenylenediamine isomers [*o*-phenylenediamine (*o*-PDA), *m*-phenylenediamine (*m*-PDA), and *p*-phenylenediamine (*p*-PDA)], with amine (–NH_2_) functionalities, have proved to be efficient heteroatom source for synthesis of N-doped CDs [3, 9, 10].

In this work, the green, blue, and orange color emitting N-doped CDs were successfully synthesized from hydrothermal treatment of ascorbic acid (AA) and individual *m*-PDA, *o*-PDA, and *p*-PDA, respectively (A*m*-, A*o*-, and A*p*-CDs, respectively). The effects of the reaction conditions and solvents, and pH of solution on the fluorescence properties of each type of CDs were systematically investigated. In particular, green color emitting CDs synthesized from ascorbic acid and *m*-PDA exhibited very high quantum yield (QY) in the ethanol solvent.

## Experimental Methods

Detailed information on the materials and instrumental analysis are described in Additional file 1: Section S1 and S2.

### Synthesis of A*m*-, A*o*-, and A*p*-CDs

To prepare A*m*-CDs, ascorbic acid (0.1 M, 0.8 mL) and *m*-phenylenediamine (0.1 M, 0.8 mL) (ratio of AA: *m*-PDA = 1:1) were added into 10.4 mL deionized water, and stirred for 5 min. Then, the mixture was transferred into a 50 mL Teflon-lined autoclave, and heated and maintained at 160 °C for 6 h in an oven for further reaction. After cooling down to room temperature (RT), the A*m*-CDs were collected after removing the suspended particles via centrifugation at 10,000 rpm for 20 min, and further purified by dialysis tube for 6 h to remove the residual chemicals. The as-obtained A*m*-CDs solution was stored at 4 °C for further characterization.

To prepare A*o*-CDs and A*p*-CDs, all experimental procedures were the same as those of A*m*-CDs, except for the precursor ratio. For A*o*-CDs, ascorbic acid (0.1 M, 1.2 mL) and *o*-phenylenediamine (0.1 M, 0.8 mL) (ratio of AA: *o*-PDA = 3:2) were used; and for A*p*-CDs, ascorbic acid (0.1 M, 0.8 mL) and *p*-phenylenediamine (0.1 M, 0.4 mL) (ratio of AA: *p*-PDA = 2:1) were used, respectively.

Additional file 1: Fig. S1 shows that the reaction temperature and the precursor ratio were optimized to obtain the highest fluorescence for each CDs.

**Fig. 1:**
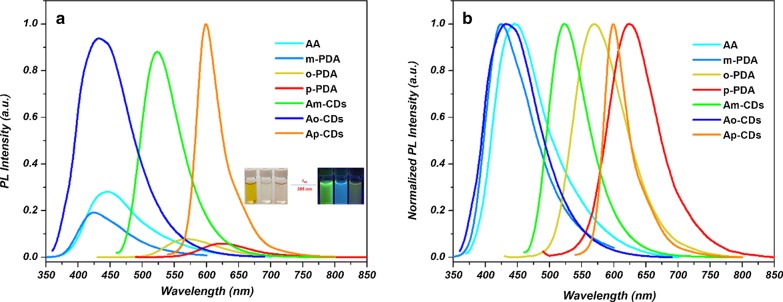
**a** Fluorescence spectra, and **b** normalized fluorescence spectra of A*x*-CDs and various precursor materials. Inset: Photographs of A*m*-CDs, A*o*-CDs, and A*p*-CDs dispersed in water under natural light (left), and under UV irradiation (*λ*_ex_ = 365 nm) (right)

Figure 1 shows that the emission intensity and wavelength of the as-synthesized CDs are totally different from those of the precursor materials. The overall comparison is summarized in Additional file 1: Table S1. It is interesting to note that green emitting A*m*-CDs can be obtained from cyan and blue emitting AA and *m*-PDA, while blue color emitting A*o*-CDs can be obtained from cyan and yellow emitting AA and *o*-PDA, which indicates the new conjugated structure formed from the reaction between AA and PDAs.

### Quantum Yield Measurement

The quantum yields (QYs) of the A*m*-, A*o*-, and A*p*-CDs were obtained by a well-known relative slope method at RT using various dyes that match their emissions with those of each CDs [9]. For A*m*-CDs (excitation wavelength of 450 nm), Rhodamine 101 in ethanol (QY = 100%) was selected as the reference; for A*o*-CDs (excitation wavelength of 360 nm), quinine sulfate (QS) in 0.1 M sulfuric acid solution (QY = 54%); and for A*p*-CDs (excitation wavelength of 514 nm), rhodamine B in water (QY = 31%).

To calculate the QYs, the integrated PL intensities of the sample and reference were plotted against absorbance at several concentrations, and the gradients were obtained and compared.

The QYs of the three CDs were obtained from the following equation:

1 $$\Phi_{{\text{s}}} = \Phi_{{\text{r}}} *\frac{{K_{{\text{s}}} }}{{K_{{\text{r}}} }}*\frac{{\eta_{{\text{s}}} }}{{\eta_{{\text{r}}} }}$$

where Φ is the relative quantum yield, *K* is the slope of the fitted line, and *η* is the refractive index of the solvent. The subscript “r” refers to the reference, and “s” to the sample. The values of refractive index for water and ethanol are 1.33 and 1.36, respectively.

## Results and Discussion

### Characterization of the As-Synthesized CDs

**Fig. 2:**
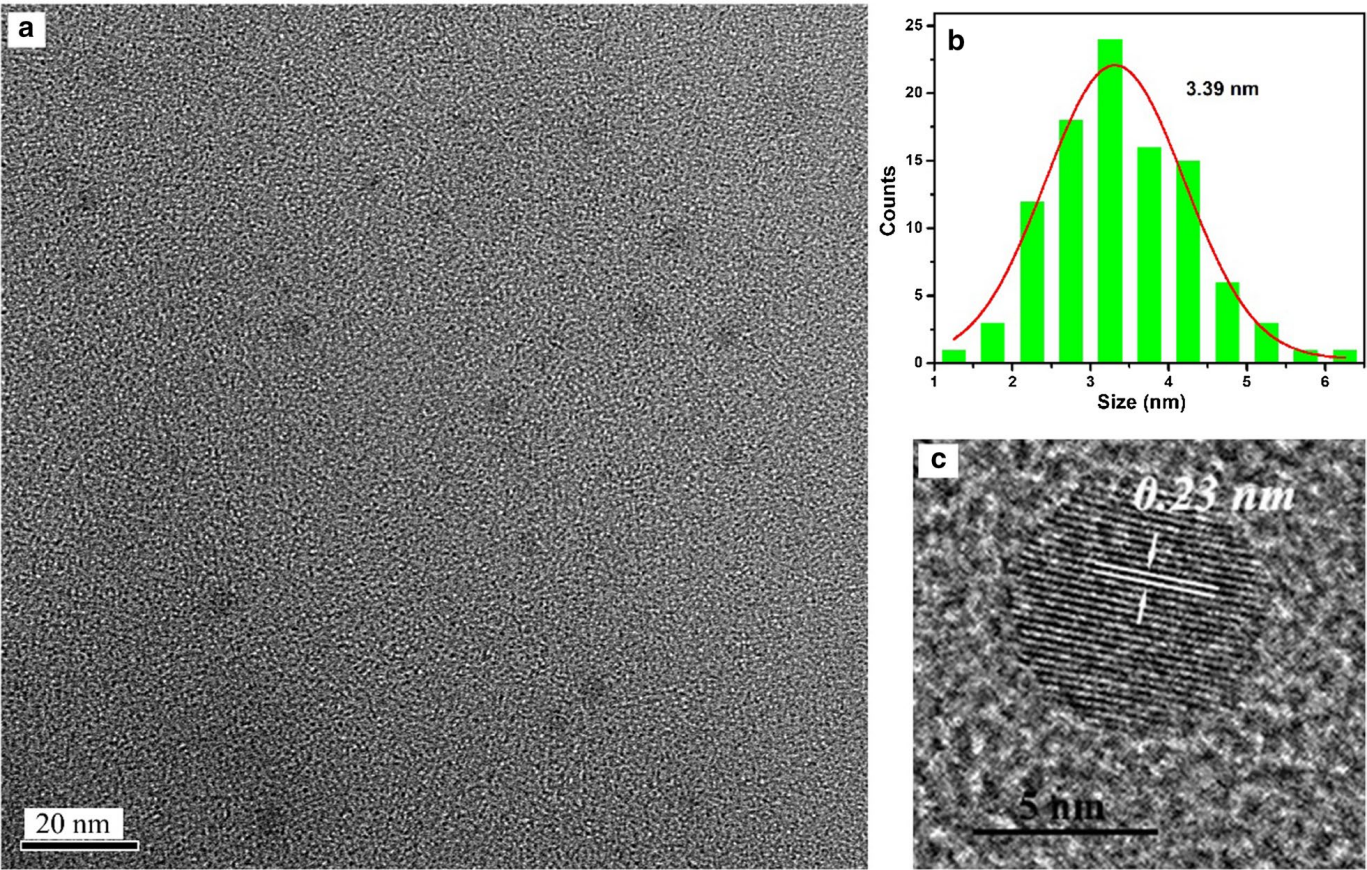
**a** TEM images of A*m*-CDs, **b** the particle size distribution histograms, and **c** HR-TEM images

**Fig. 3:**
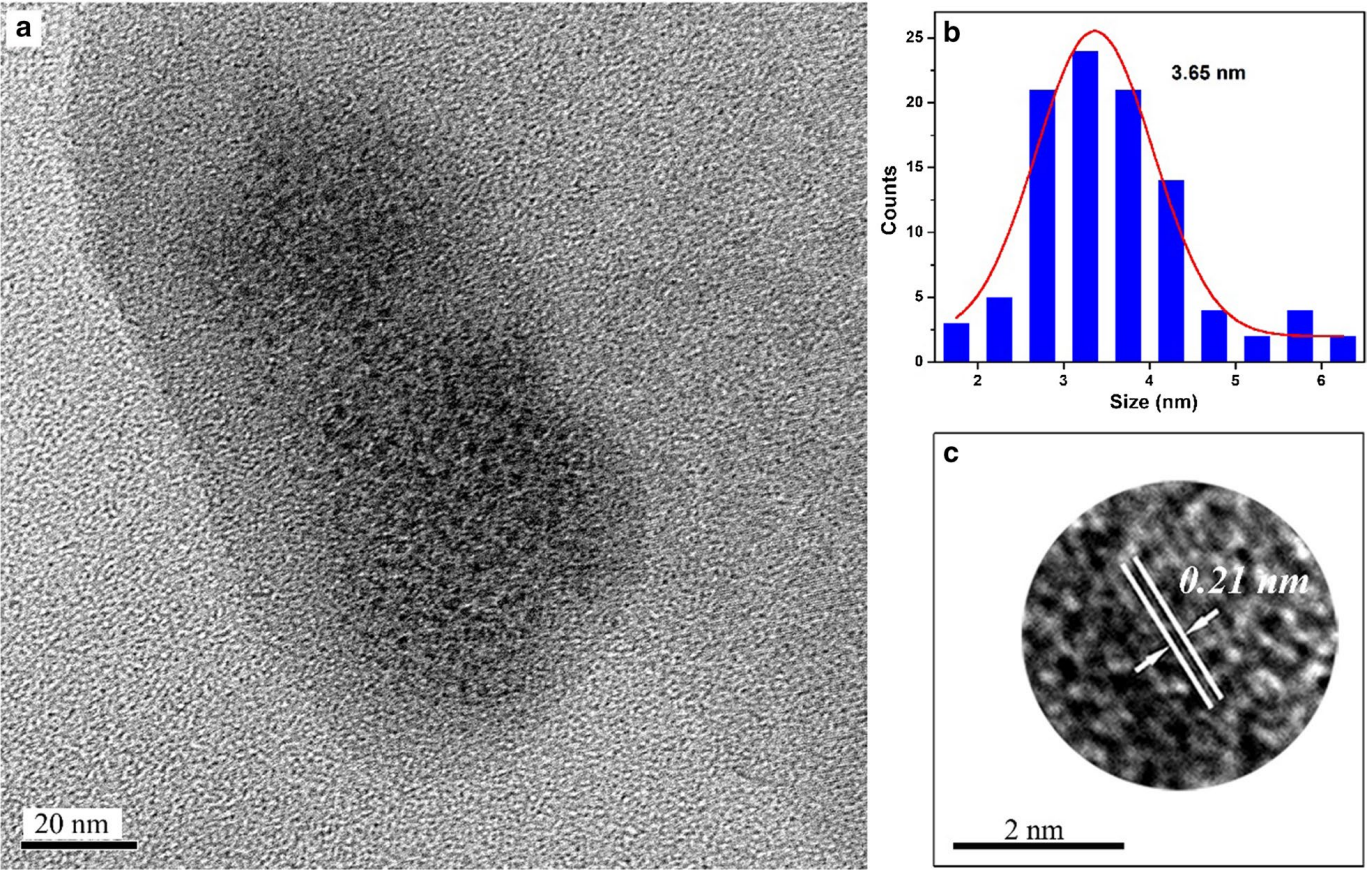
**a** TEM images of A*o*-CDs, **b** the particle size distribution histograms, and **c** HR-TEM images

**Fig. 4:**
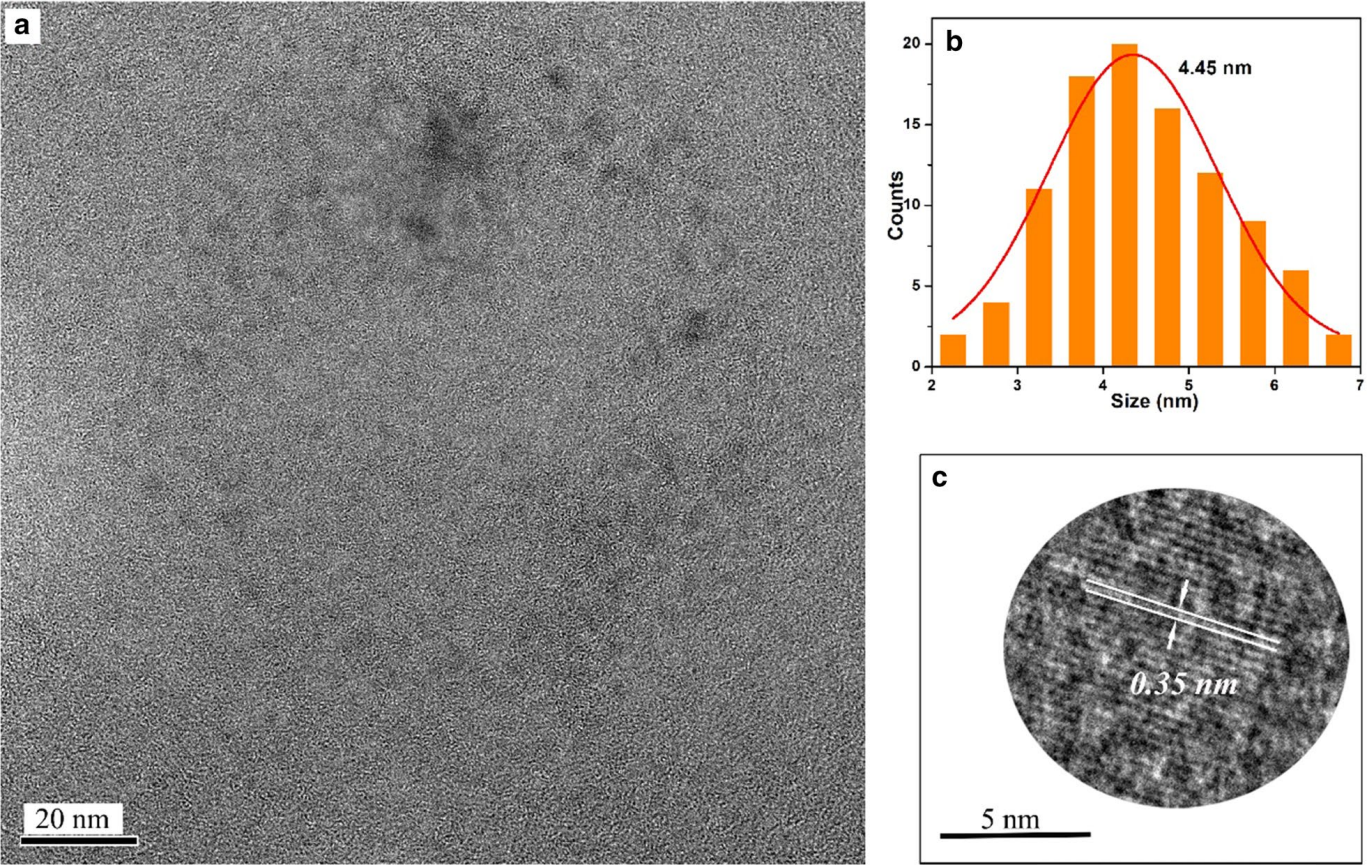
**a** TEM images of A*p*-CDs, **b** the particle size distribution histograms, and **c** HR-TEM images

The morphology and size of the A*x*-CDs (*x* = *m*, *o*, and *p*) were analyzed from TEM images. Figures 2, 3, and 4 show that the mean diameters of A*m*-CDs, A*o*-CDs, and A*p*-CDs were 3.39 nm, 3.65 nm, and 4.45 nm, respectively. The interplanar spacings of A*x*-CDs were 0.23 nm, 0.21 nm, and 0.35 nm analyzed from HR-TEM images, respectively, which correspond to the (100) and (002) planes of graphite carbon [11].

**Fig. 5:**
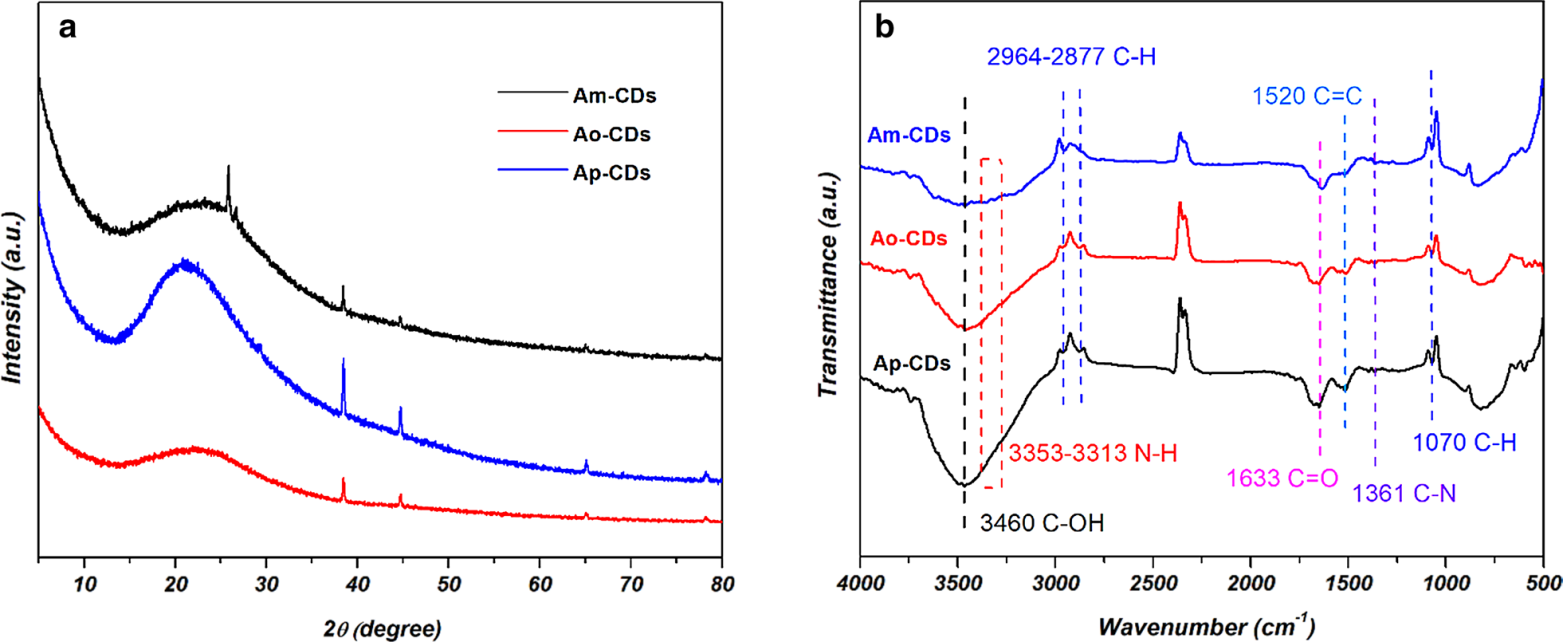
**a** XRD patterns, and **b** FTIR spectra of A*x*-CDs

The crystal structures of the A*x*-CDs were investigated by XRD. Figure 5a shows that the three CDs have a broad single diffraction peak around 2*θ* = 21°–23°, which originates from graphitic carbon structure [3, 12].

The chemical bonds and surface functional groups of A*x*-CDs were analyzed by FT-IR spectra. Figure 5b shows the peaks at ~ 3460 and ~ 3313–3353 cm^−1^ that can be attributed to the stretching vibrations of O–H and N–H, respectively. The presence of hydrophilic groups can improve the solubility of CDs in polar solvent by the formation of hydrogen bonding [13, 14]. The peaks at ~ 1070, ~ 2877 and ~ 2964 cm^−1^ can be assigned to the stretching vibrations of C–H [8]. The strong peak observed at ~ 1633 cm^−1^ can be ascribed to the stretching vibration of C=O bond in the amide group, which confirms the amidation reaction between the carboxylic acids of AA and amines of PDAs [15]. The peaks that appear at ~ 1520 cm^−1^ can originate from the bending vibration of C=C [16]. In addition, the peaks observed at ~ 1361 cm^−1^ can be ascribed to the stretching vibration of C–N, which confirms the presence of nitrogen atom in the as-synthesized CDs [10]. The near identity of the FT-IR spectra of all three CDs indicates the presence of similar chemical bonds and functional groups on the CDs, regardless of the position of amine group in PDA isomers species.

**Fig. 6:**
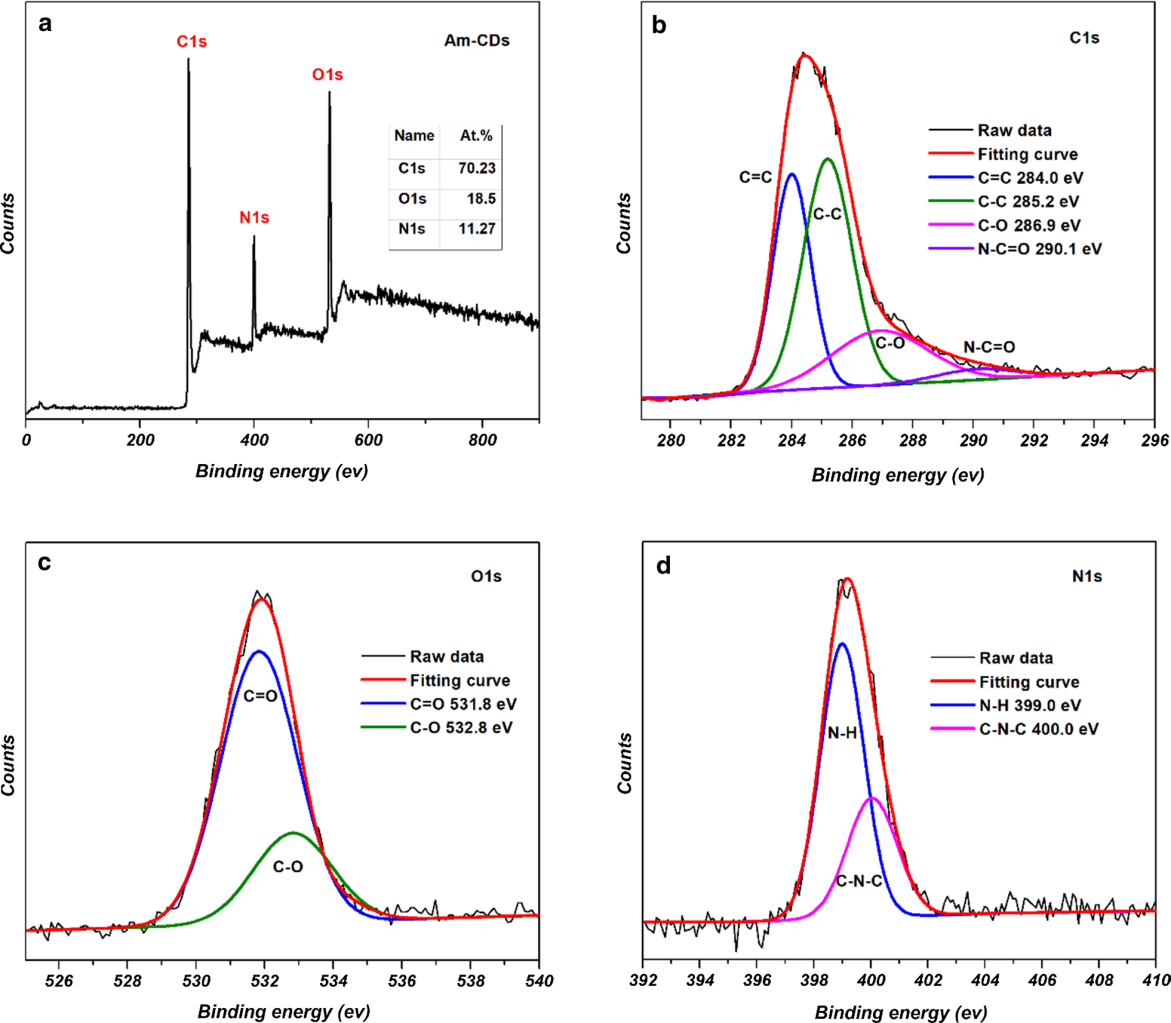
**a** XPS survey spectrum of A*m*-CDs. The high resolution **b** C1*s*, **c** O1*s*, and **d** N1*s* XPS spectra of A*m*-CDs

XPS was used to analyze the elemental composition and functional groups of the A*x*-CDs. Figure 6a shows the XPS survey spectrum of A*m*-CDs, which indicates the existence of C, O, and N atoms in the synthesized A*m*-CDs. Additional file 1: Figs. S2 and S3 show that the three CDs have similar elemental compositions, as summarized in Table 1. The XPS analyses also indicate similar oxidation state and functionalities in the three CDs. Figure 6, and Additional file 1: Figs. S2 and S3 show the high-resolution C1*s* XPS spectra for A*x*-CDs, which reveal that carbon can be deconvolved into several peaks centered at ~ 284.0, ~ 285.2, ~ 286.9, and ~ 290.1 eV, which correspond to C=C, C–C, C–O, and N–C=O groups, respectively. The high-resolution O1s spectra can be deconvoluted into peaks shown at ~ 531.8 and ~ 532.8 eV that can be attributed to C=O and C–O groups, respectively [17]. The N1*s* spectra reveal the presence of N–H, C–N–C, and graphitic N groups shown at ~ 399.0, ~ 400.0, and ~ 401.4 eV, respectively [18].

**Table 1:** The elemental compositions of A*x*-CDs

Sample	C (%)	O (%)	N (%)
A*m*-CDs	70.23	18.50	11.27
A*o*-CDs	69.30	21.85	8.85
A*p*-CDs	70.63	20.28	9.09

### Optical Properties of the A*x*-CDs

**Fig. 7:**
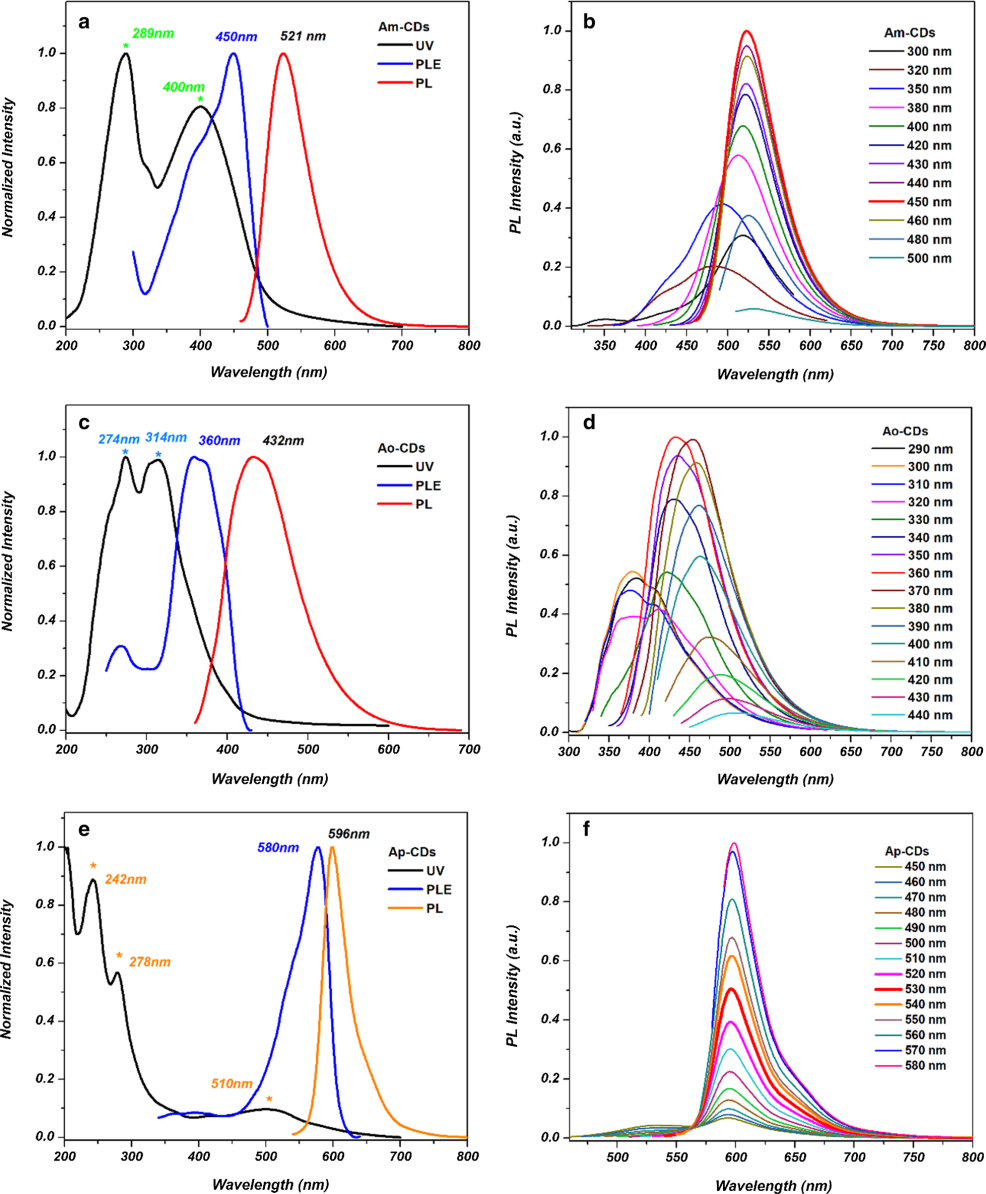
The normalized UV–Vis absorption spectra, PL excitation, and PL emission spectra of the **a** A*m*-CDs, **c** A*o*-CDs, and **e** A*p*-CDs. The normalized PL emission spectra of the **b** A*m*-CDs, **d** A*o*-CDs, and **f** A*p*-CDs at different excitation wavelengths

The optical properties of the A*x*-CDs were explored by the UV–Vis absorption and PL spectra. Figure 7 shows the UV–Vis absorption, photoluminescence excitation (PLE), and PL spectra of the A*x*-CDs. Two absorption peaks centered at 289 and 400 nm are observed in the UV–Vis absorption of A*m*-CDs (Fig. 7a), which correspond to the *π*–π* transitions of the C=C structure, and the *n*–π* transitions of C=O groups [15]. A*o*-CDs and A*p*-CDs showed two peaks in the UV–Vis spectra, however the peak positions and intensities were different (Fig. 7c, e). This difference might be attributed to the different extent of electronic transitions. Moreover, the additional broad absorption peak shown at ~ 510 nm can be attributed to the surface absorption of the A*p*-CDs, and succedent excitation of the PL emission [19]. Accordingly, the PLE and PL spectra are different for all three A*x*-CDs. The A*m*-CDs show emission in the green region at 521 nm when excited at 450 nm. The A*o*-CDs and A*p*-CDs show excitation peaks at 360 and 580 nm and emit at blue region at 432 nm and orange region at 596 nm, respectively.

Figures 7b, d, f show that A*m*- and A*o*-CDs show excitation-dependent emission while A*p*-CDs show excitation-independent emission. The excitation wavelength-dependent PL emission behavior might originate from the nonuniform CDs size, and presence of various surface defects, and various surface functional groups in the CDs [20, 21]*.* The excitation wavelength-independent PL emission behavior of A*p*-CDs indicates uniform emission states, which also result in narrow emission width. The different excitation wavelength related PL properties among the A*x*-CDs imply the different energy states, and their morphology [22, 23].

### Solvent Effects and QY on the PL Emission Properties

The effects of solvent, including deionized water (H_2_O), Methanol (MeOH), Ethanol (EtOH), Isopropyl alcohol (IPA), Acetone (ACE), Acetonitrile (ACN), *N*,*N*-Dimethylformamide (DMF), and Dimethyl sulfoxide (DMSO) on the PL emission properties of the A*x*-CDs were investigated. Additional file 1: Fig. S4 shows that the PL emission wavelength changes at different solvents. This shows the typical solvatochromic properties of CDs caused by the interaction between surface functional groups of CDs and solvents [21, 24].

Additional file 1: Fig. S5 shows that the A*m*-CDs possessed the highest QY among the three CDs. In addition, the A*x*-CDs in ethanol solvent exhibit higher QY than those in water, which can be explained by (1) higher extent of agglomeration of CDs in high polar solvent, (2) increased rate of non-radiative decay during the interaction between highly polar solvent and CDs, and (3) water-induced morphological change [25].

### pH Effects on the Fluorescence Emission of A*x*-CDs

**Fig. 8:**
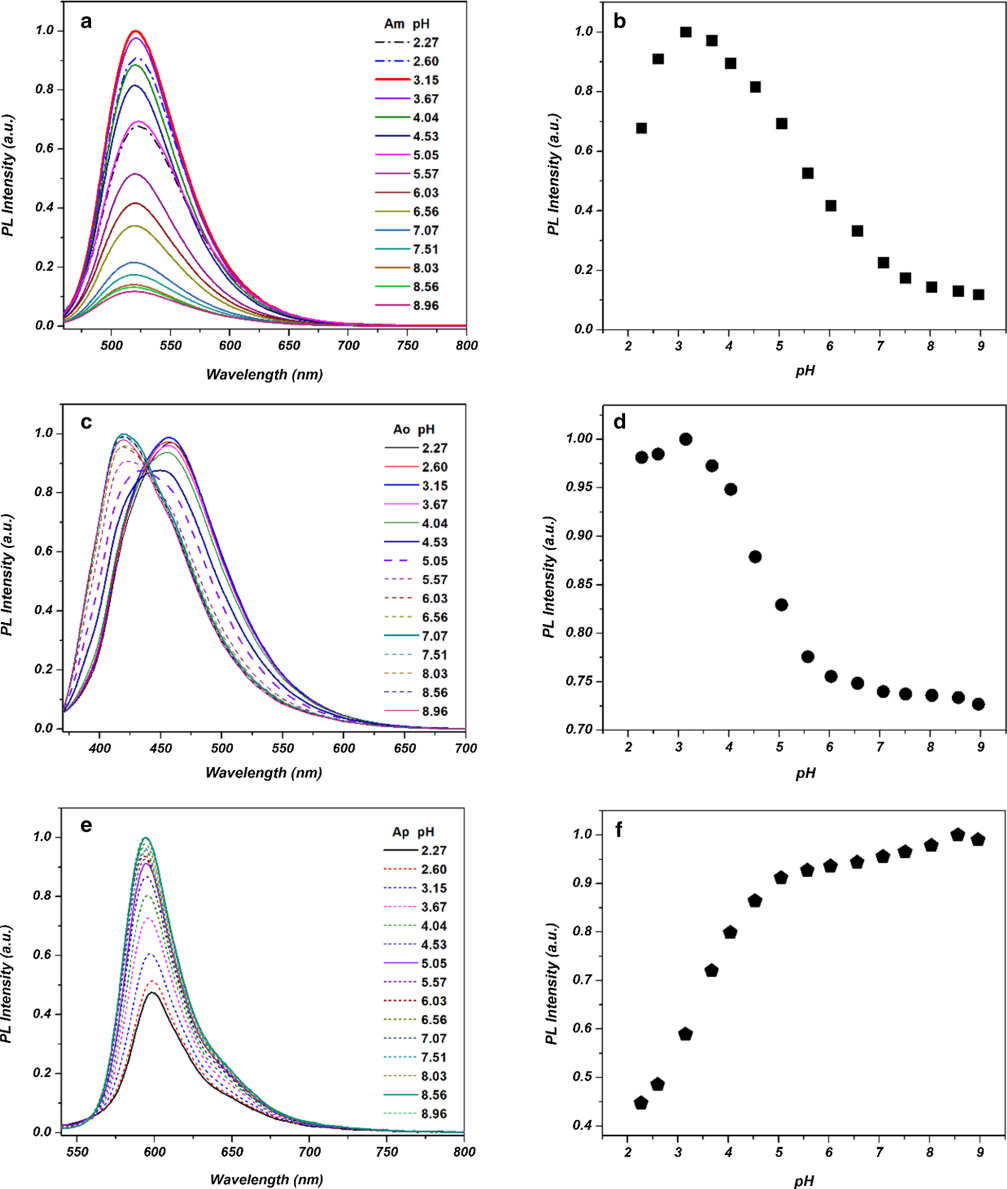
PL emission and intensity change of **a**, **b** A*m*-CDs, **c**, **d** A*o*-CDs, and **e**, **f** A*p*-CDs under various pH conditions

The PL emission intensities of the as-prepared A*m*-, A*o*-, and A*p*-CDs were monitored at various pH conditions. Figure 8 shows that A*m*- and A*o*-CDs exhibit similar PL emission behavior as the pH of solution changes. The decrease in PL emission as the pH increases can be attributed to the deprotonation of the surface functional groups of the A*m*- and A*o*-CDs, resulting in the agglomeration of CDs [26–29].

On the other hand, for A*p*-CDs, the PL intensity increases as the pH of solution increases. This phenomenon can be attributed to the different surface charge of A*p*-CDs from the other CDs.

**Fig. 9:**
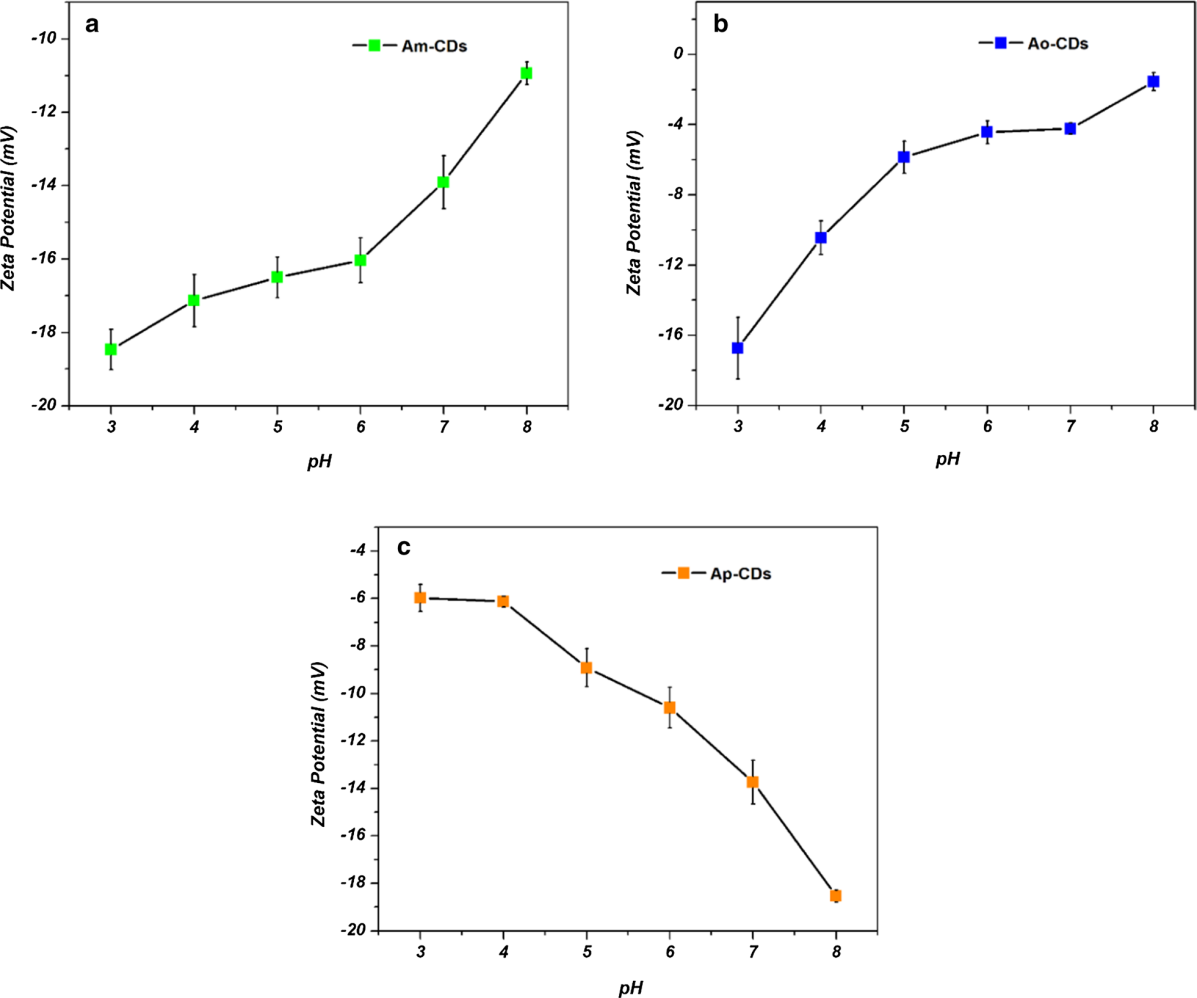
Zeta potential of **a** A*m*-CDs, **b** A*o*-CDs, and **c** A*p*-CDs under various pH values, respectively

To investigate the different pH-dependent behaviors between A*x*-CDs, the zeta potential was monitored at various pH values. As shown in Fig. 9, the zeta potentials of the A*m*- and A*o*-CDs gradually decreased with increasing pH, whereas the zeta potential of A*p*-CDs increased with increasing pH. This might result in lesser agglomeration and enhanced the PL intensity of A*p*-CDs [30, 31].

## Conclusion

In this work, the green, blue, and orange color emitting N-doped CDs have been successfully synthesized from the reaction between ascorbic acid (AA) and *m*-PDA, *o*-PDA, and *p*-PDA, respectively. For this purpose, a simple low temperature hydrothermal synthesis method has been employed. The photophysical and optical properties of the three CDs have been investigated thoroughly at different solvents and pH. The as-synthesized A*x*-CDs exhibited higher QYs in ethanol than that in water. The lesser agglomeration, reduced rate of non-radiative decay, and lesser morphological change of CDs might be the reason behind such behavior. In addition, the surface charge of synthesized A*x*-CDs resulted in different pH-dependent PL emission properties. These unique properties of the as-synthesized CDs will enable their applications in different fields of imaging and sensing.

## Supplementary information

**Additional file 1**. Supplementary Information.

## Data Availability

All data generated or analyzed during this study are included within this article and its supplementary information files.

## Abbreviations

CDs: Carbon dots
AA: Ascorbic acid
*m*-PDA: *m*-Phenylenediamine
*o*-PDA: *o*-Phenylenediamine
*p*-PDA: *p*-Phenylenediamine
A*x*-CDs: *x* = *m*, *o*, And *p*
QY: Quantum yield
SI: Supplementary Information
RT: Room temperature
QS: Quinine sulfate
PLE: Photoluminescence excitation
H_2_O: Deionized water
MeOH: Methanol
EtOH: Ethanol
IPA: Isopropyl alcohol
ACE: Acetone
ACN: Acetonitrile
DMF: *N*,*N*-Dimethylformamide
DMSO: Dimethyl sulfoxide
HR-TEM: High-resolution transmission electron microscopy
FT-IR: Fourier transform infrared spectroscopy
XRD: X-ray diffraction
XPS: X-ray photoelectron spectroscopy
