# Supplementary material for: Multicolor Emitting N-Doped Carbon Dots Derived from Ascorbic Acid and Phenylenediamine Precursors
Source: Nanoscale Res Lett. 2020 Dec 3;15:222. doi: 10.1186/s11671-020-03453-3 (PMC7714885; doi:10.1186/s11671-020-03453-3)
Supplement: Supplementary file 1 — Additional file 1. Supplementary Information. [file 11671_2020_3453_MOESM1_ESM.docx]

**Supplementary Information**

**Multicolor emitting N-doped carbon dots derived from ascorbic acid and phenylenediamine** **precursors**

Linlin Wang, Won Mook Choi, Jin Suk Chung and Seung Hyun Hur*

School of Chemical Engineering, University of Ulsan, Daehak-ro 93, Nam-gu, Ulsan 44610, Republic of Korea

*E-mail: shhur@ulsan.ac.kr

**1. Materials**

Ascorbic acid (AA), m-phenylenediamine (m-PDA), o-phenylenediamine (o-PDA), p-phenylenediamine (p-PDA), rhodamine 101, quinine sulfate (QS) and rhodamine B were purchased from Sigma-Aldrich Co. (USA). All chemicals were analytical grade reagents and used as purchased without further purification. The deionized water with a resistivity of 18.2 MΩ was used in all experiments.

**2. Characterizations**

The fluorescence of the samples was recorded using a Cary Eclipse fluorescence spectrophotometer (Agilent Technologies, USA) using a 1.0 cm quartz cuvette. The absorption study of the samples was performed with the help of a UV-Vis spectrometer (UV-Vis, SPECORD 210 PLUS, Analytik Jena, Germany). The analysis of functional groups was done using the Fourier Transform Infrared (FTIR, Nicolet iS5, Thermo Fisher Scientific, USA) spectroscopy. X-ray photoelectron spectroscopy (XPS, Thermo ESCALAB 250 Xi, Thermo Fished Scientific, USA) using Al Kα X-ray radiation (1486.6 eV) was used to determine the oxidation states of the constituent elements. X-ray diffraction (XRD, Rigaku D/MAZX 2500V/PC model, Japan) was carried out using Cu Kα radiation (40 kV, 30 mA, λ = 1.5415 Å) at a scan rate of 2° min^−1^ over the 2θ range of 10-100°. The size and morphology of the as-synthesized particles were monitored by high-resolution transmission electron microscopy (HR-TEM, JEM-2100 F, JEOL, Japan, operating voltage 200 kV). Zeta potential measurements was carried out using Malvern Nano ZS instrument (Worcestershire, UK).


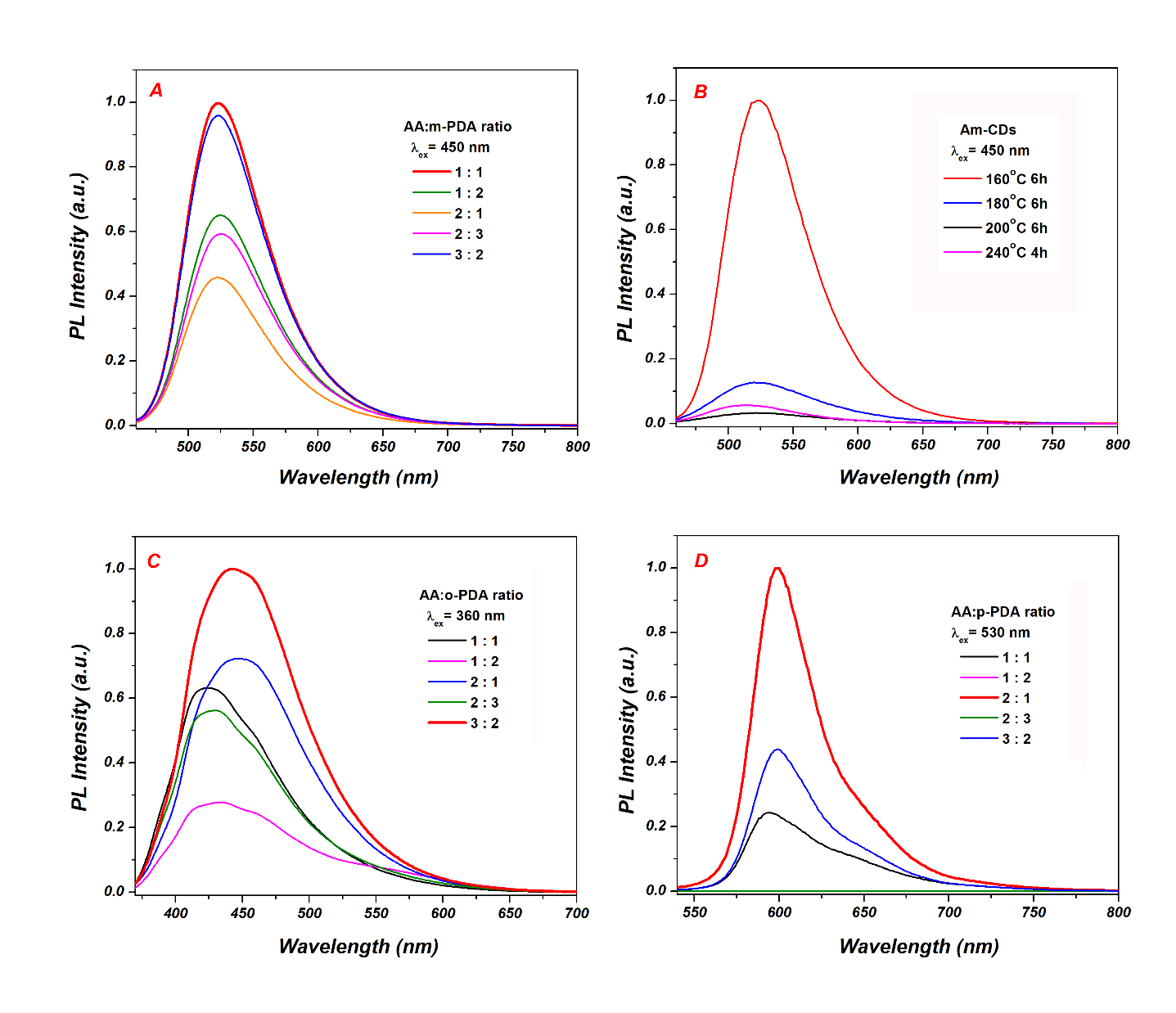


**Fig. S1.** (A, B) Fluorescence spectra of Am-CDs at different ratio, and different reaction temperature and time from AA and m-PDA. (C) Fluorescence spectra of Ao-CDs at different ratio from AA and o-PDA. (D) Fluorescence spectra of Ap-CDs at different ratio from AA and p-PDA.

**Table S1.** The excitation wavelength, emission peak wavelength and fluorescence color of different CDs.

| **CDs raw materials** | **λ_ex_/nm** | **Emission peak/nm** | **Fluorescence color** |
| --- | --- | --- | --- |
| **AA** | 360 | 446 | Blue |
| **m-PDA** | 310 | 425 | Blue |
| **o-PDA** | 420 | 569 | Yellow-green |
| **p-PDA** | 480 | 624 | Red |
| **AA + m-PDA** | 450 | 524 | Green |
| **AA + o-PDA** | 360 | 432 | Blue |
| **AA + p-PDA** | 530 | 599 | Orange |


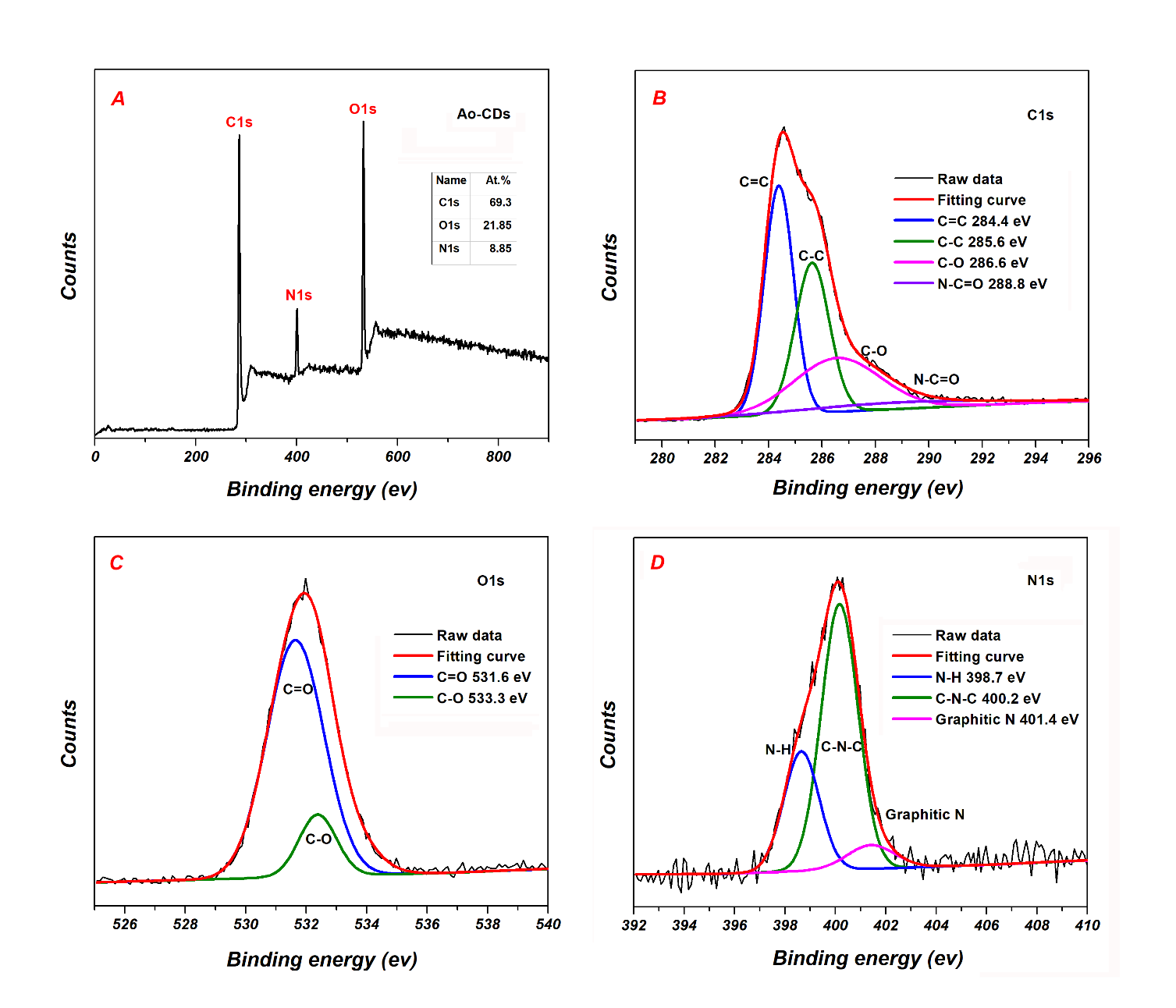


**Fig. S2.** (A) XPS survey spectrum of Ao-CDs. The high resolution (B) C1s, (C) O1s, and (D) N1s XPS spectra of Ao-CDs.


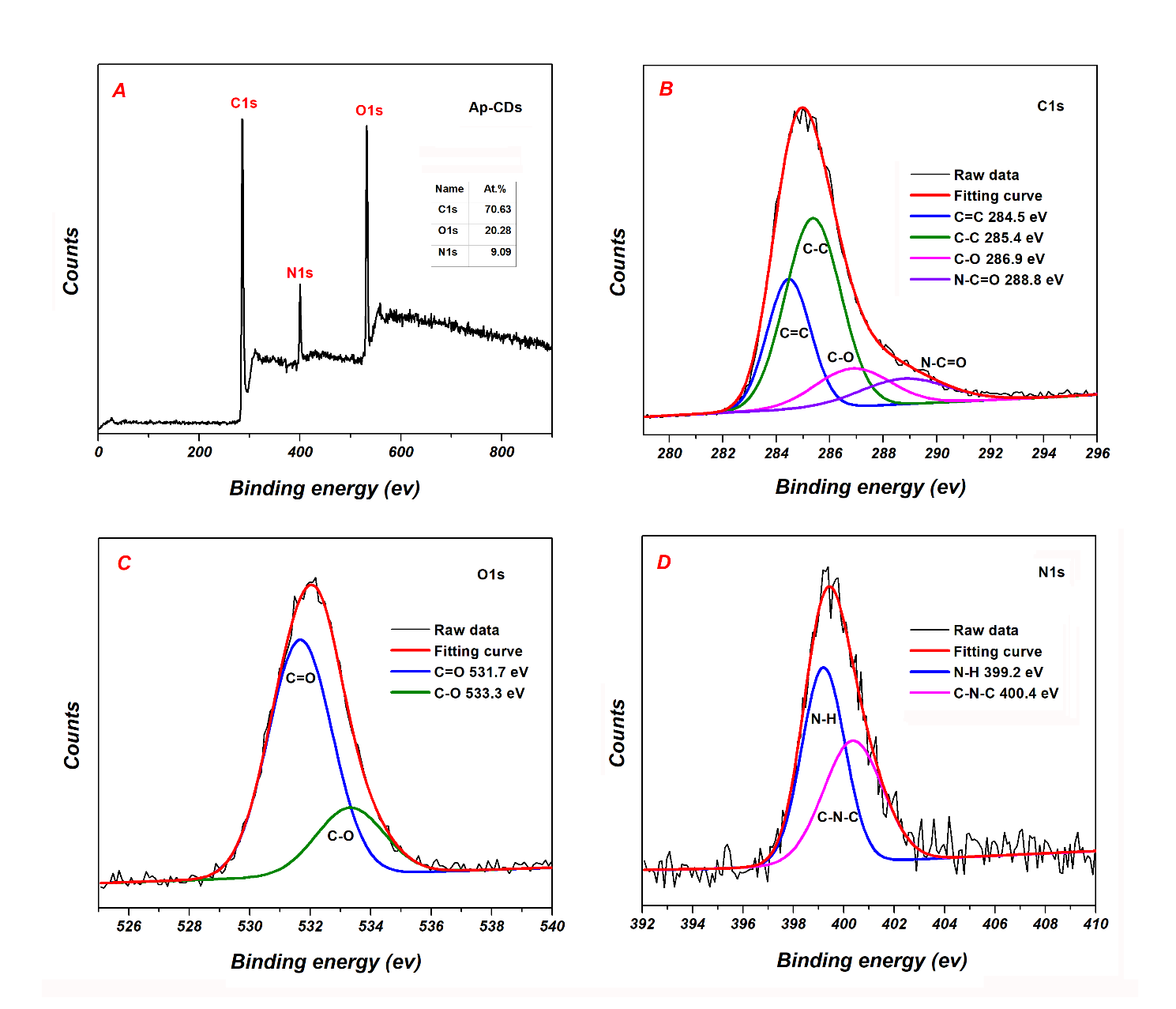


**Fig. S3.** (A) XPS survey spectrum of Ap-CDs. The high resolution (B) C1s, (C) O1s, and (D) N1s XPS spectra of Ap-CDs.


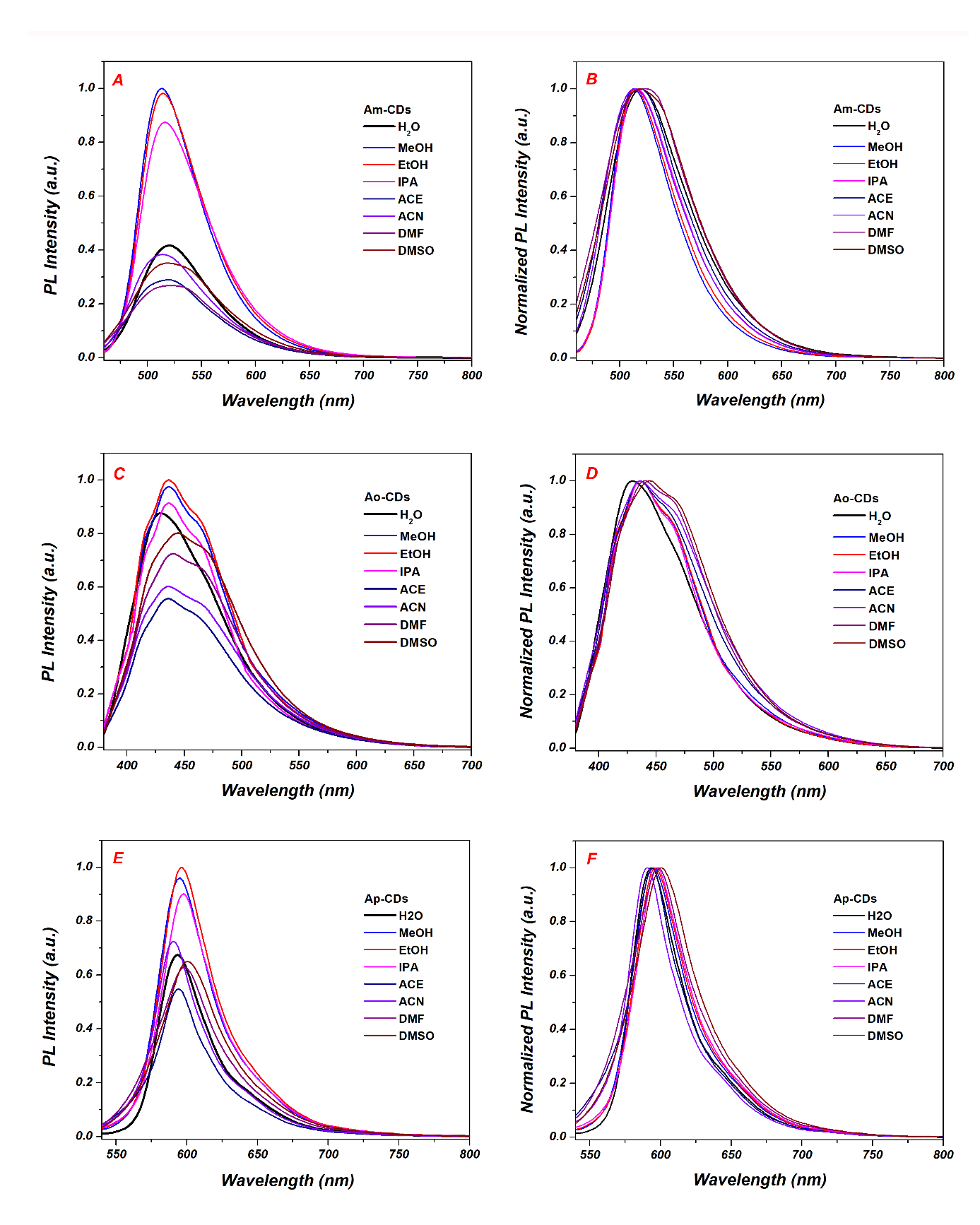


**Fig. S4.** The effect of different solvents on PL emission properties of (A, B) Am-CDs, (C, D) Ao-CDs and (E, F) Ap-CDs.


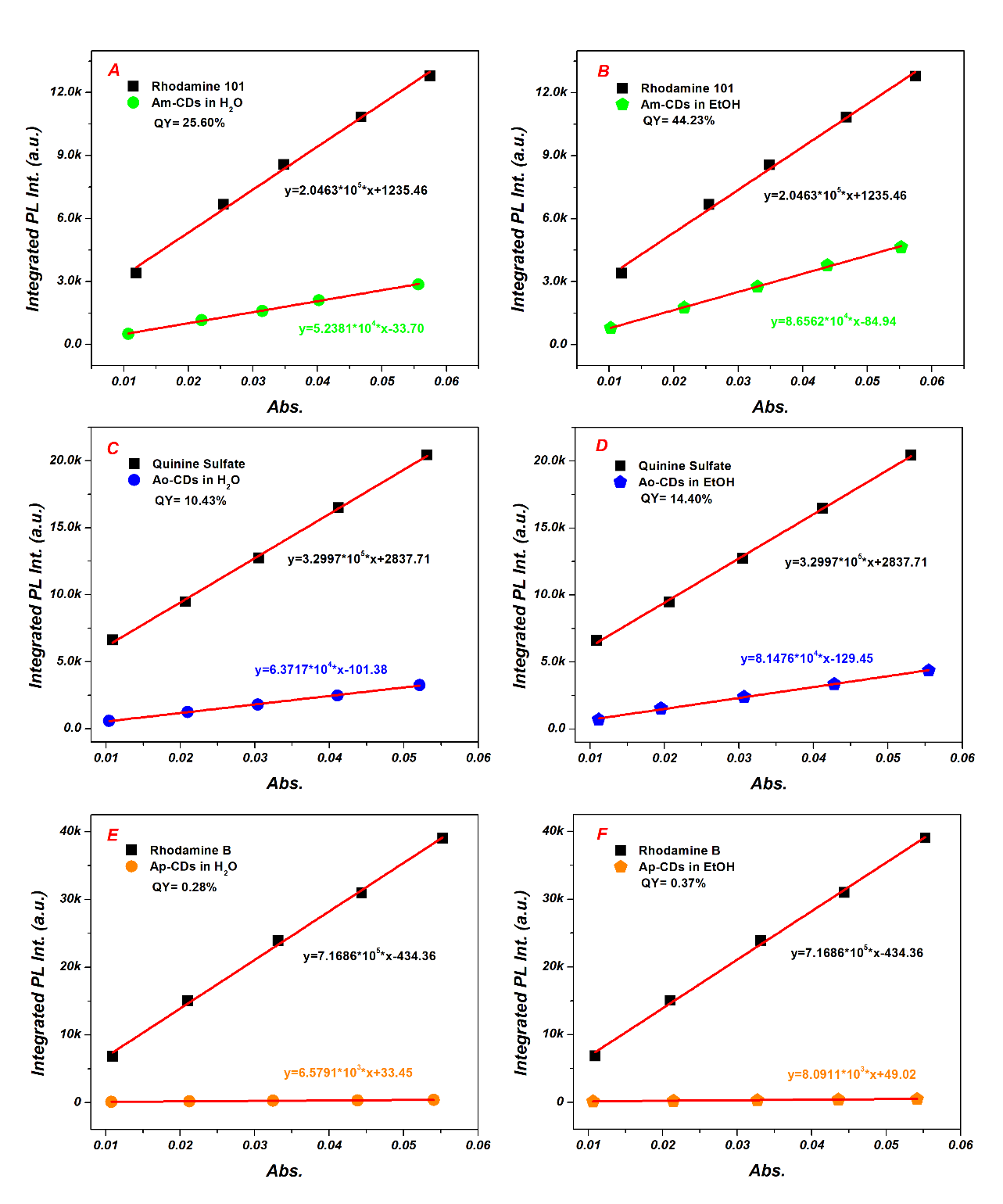


**Fig. S5.** Plots of integrated PL intensity of Am-CDs and rhodamine 101 as a function of optical absorbance at 450 nm in (A) water and (B) ethanol, respectively. Plots of integrated PL intensity of Ao-CDs and quinine sulfate as a function of optical absorbance at 360 nm in (C) water and (D) ethanol, respectively. Plots of integrated PL intensity of Ap-CDs and rhodamine B as a function of optical absorbance at 514 nm in (E) water and (F) ethanol, respectively.
